# Dr. Huggan, on the Effects of Venesection and Opium

**Published:** 1799-10

**Authors:** A. Huggan

**Affiliations:** Ashburton


### *To the Editors of the Medical and Physical Journal.*

GENTLEMEN,

HAVING, by accident, a few days ago, met with the last Number of your Journal, I shall venture, through so respectable a channel, to lay before the Public any observations on professional subjects which I may be enabled to make. Satisfied that a Regimental Hospital, if properly conducted, is one of the best schools in the world for acquiring practical knowledge, I have endeavoured to improve the advantages of my situation to the utmost. I have been upwards of six years surgeon of the western regiment of Kentish militia, during which time our number of sick has never been inconsiderable; whereby much opportunity of practice has been afforded me. I have been in the habit of keeping a Journal of the different cases as they occurred, wherein I carefully noted every symptom of which a patient complained, the various remedies exhibited, the time when, and with what view given. I also marked every change that took place in the course of a disease, and the effect of the medicines made use of; and lastly, my own opinion of the method of cure which I had adopted. In the course of my practice, I have endeavoured, on every occasion, to determine the justness of pre-conceived theories, by experience, and on every subject to think for myself, uninfluenced by the "tenets of the schools," or the opinions of others. I have had an opportunity of giving every new remedy a fair and candid trial, and in every instance wherein my present practice or opinions differ from what are generally adopted, I can solemnly assure you, that they are the result of actual experience only, for I have no theory to serve. The prevalence of any mode of practice is certainly not a clear proof of its being useful; nor is it a sufficient recommendation, that it may be practised with safety; if it is not evidently beneficial, it ought to be laid aside.

In this light I consider the too general custom of bleeding, as a means of cure in febrile diseases, contusions, &c. which I have no hesitation in asserting, is not necessary in any complaint with which we are acquainted. If we grant, that every *deviation from the healthy state denotes debility, either general or partial,* surely whatever has a tendency to debilitate further, it is reasonable to suppose, ought to be carefully avoided. It certainly cannot be denied that, in every disease wherein bleeding has been used, complete recovery has been protracted, owing to the debility thereby occasioned. We are directed to use blood-letting to lessen irritability, to take off the phlogistic diathesis, to deplenish the blood vessels, and to prevent inflammation. I know from much experience, that these indications can be fulfilled as expeditiously, as effectually, and certainly with much more safety by opium, if given in a dose proportioned to the violence of the disease which we have to combat. The timidity of practitioners has brought opium into discredit, as a means of cure in the diseases alluded to. Though the ill effects of the loss of blood, unless excessive, are seldom perceivable in youth, yet they rarely fail of being felt before the age of forty-five. People who have been often bled when young, about this period of life generally begin to be affected with chronic pains; they recover very slowly from fits of illness, and are liable to febrile paroxysms, that become more teazing than dangerous, and in which there is seldom any increase of irritability. I have rarely been deceived in my conjectures respecting patients of this description, when I have met with them. The first of the cases mentioned in the last number of the Medical and Physical Journal, by Dr. Denman, shows that bleeding does not always prevent inflammation or abortion. Nor is it clearly proved, that by taking away blood, we lessen the diameter of the blood vessel, as we find that six ounces from a large orifice has a greater effect than twenty from a small one.

I have taken the liberty of subjoining a short history of a few cases, as proofs of what I have advanced, respecting the use of blood-letting and opium, and with what safety and advantage a large dose of it may be given, when necessary.

Case i.—R. A. about thirty years of age, was on the 9th of August, 1794, brought to the regimental hospital, on account of a blow which he had received on the right side of his head with a poker; for which he was bled twice, and took several doses of salts, by which means, the pain of the head, which seemed very violent, was removed; yet he was unable to go to his duty before the beginning of the following November. This man, on on the evening of the King's birth-day, 1796, received on the left side of his head a severer blow, with a similar weapon. He now complained of pain not only on the affected side of his head, but also on the other. Bleeding was omitted, and he had given him three grains of powdered opium, which so far mitigated the violence of the symptoms, that he fell asleep. It was necessary to give him an opiate for several succeeding evenings, as the pain returned more than once, during the first week, but was not very severe. He took only a little calomel, and a dose of salts, besides the opium, and was able to go to duty within five weeks after the accident.

Case 2.—J. S. aged forty, came into the Hospital, in Nov. 1795, for a blow on the thorax, which rendered respiration very painful. Twelve ounces of blood taken from a large orifice gave him no relief.

Case 3.—W. M. about twenty-seven years old, was admitted into the Hospital in October 1796, for a pain in the region of the stomach, with nausea; he had been costive during the three preceding days. A dose of salts was given him which gave him stools, but did not in the smallest degree abate the pain. He felt some relief from a blister put to the pained part; but on the third day he found the pain gradually increasing, and towards evening it became very violent, with vomiting, quick pulse, dry skin, &c. the blistered part also was quite dried up. A draught, with two hundred drops of laudanum, of which he was directed to take one third every two hours till he was easier; a large blister was prepared for him, and was applied to the region of the stomach. He was relieved soon after he took the second dose of the opiate, but did not sleep much during the night. By this means, though the pain was greatly abated, yet it was not removed entirely for a week, which rendered a repetition of the opiate (but not in such a dose as the former) necessary, every evening during the period.

In this case, was the seat of the pain in the stomach, or in the gall-ducts?

Case 4.—T. B. twenty-eight years of age, had just recovered from a fever when the regiment was ordered from Portsmouth in July, 1797. While he was unloading a baggage-waggon at Maidstone, he was struck on the back by a large chest of immense weight. I saw him about twenty minutes after the accident, when he appeared to be in great pain, which was increased by the least motion. I gave him immediately ninety drops of laudanum, which not affording him any relief, was repeated two hours afterwards, whereby he soon fell asleep; in the evening he took other sixty drops. The following being a halting day, a dose of salts was given him, and an opiate draught, with sixty drops of laudanum, was directed for him at bed-time, but was afterwards not judged necessary; it was therefore not taken. Next day he was able to travel in a waggon without any inconvenience, nience; the pain in his back, from the blow, being now confiderably relieved. He went to his duty about a fortnight after the accident.

Case 5.—Corporal M. aged twenty-five, very ftout, of a florid complexion, was in February, 1798, feized with pains in the thorax, painful refpiration, and other fymptoms of pneumonia, with a quick and full pulfe. He took immediately two pills containing five grains of calomel, and three of powdered opium; about two hours afterwards, as he was not relieved, other two grains of opium were given him, and blifters put to the pained parts, which gave him eafe, though he paffed a fleeplefs night. Two grains of opium were given him the three fucceeding evenings; and without the ufe of any other remedy he foon recovered.

Case 6.—G.B. a ftout young man, aged twenty-three years; was in April, 1798, thrown with great force againft a heap of bricks, which he ftruck with his head. I faw him about an hour afterwards, when I found on the right fide of his head a flight wound; the contufed parts much fwelled, with pains darting through the whole head, and which feemed to be excruciating; the blood flowed from his mouth and right ear: altogether this was truly an alarming cafe. I gave him immediately a draught with 120 drops of laudanum, which it was neceffary to repeat about two hours afterwards; foon after this fecond dofe he fell into a profound fleep, which lafted upwards of feven hours. It was judged requifite to give him at bed-time the fame evening another draught, with fixty drops of laudanum, as the pains, &c. were not fo much abated as I expected. On the following day he had a brifk cathartic given him, and on that, and four fucceeding evenings, he took an opiate confifting of one hundred drops of laudanum; when all the fymptoms, except the fwelling and wound, were removed. As he was an officer's fervant, and comfortably lodged, he was not taken into the hofpital, therefore I was uncertain how foon afterwards he recovered entirely.

I fhall add two other cafes, to fhow the good effects of opium in complaints where laxatives feem indicated.

Case 7.—A gentleman of high rank, upwards of forty years of age, and very ftout, was in November laft feized with a fevere pain in the right fide of the abdomen, which grew worfe in the courfe of the day, till it became quite alarming. After repeated attempts to procure ftools by laxatives and glyfters of tartarifed antimony and Glauber's falts, without effect, towards evening I gave him ninety drops of laudanum in a little fyrup and water, which dofe was repeated within half an hour, and foon after followed a copious evacuation by ftool, which gave him relief. As the pain was not entirely removed, removed, a large blifter was put to the affected part. A few days afterwards, when the pain had entirely left him, he had a fit of gout which came on very flowly, and very unlike a firft attack of that complaint; he had no degree of fever, though the pain in both great toes was fevere. This gentleman had in his younger days been afflicted with pneumonia, and fome other complaints, for which bleeding was judged neceffary; he had been fome years fubject to flying pains, fpafms of the calves of his legs, and occafionally a weaknefs of ftomach, for which he had at different times taken fteel and other tonics.

Case 8.—Corporal B. was one evening, on our paffage from Ireland, feized with all the fymptoms of colic, in a degree more violent than I had ever witneffed it; he took ninety drops of laudanum, and about fix drachms of tincture of fenna. Within an hour he had feveral loofe ftools, whereby he was greatly relieved, and a few days afterwards was quite well.

The fubjects of the above cafes are ftill living, and free from any complaint whatever.

In my inaugural differtation on the the epidemic catarrh, publifhed at Edinburgh, in June, 1793, I endeavoured, in fome degree, to combat the too frequent ufe of the lancet in that and fome other difeafes of the acute kind; but not with the fame confidence that I now endeavour to do fo. On this, as well as other profeffional fubjects, I gladly embrace this public opportunity of acknowledging my obligations to Dr. Lubbock, of Norwich.

I fhall mention the following cafe, as I wifh it to be generally known.—By the inattention of one of the hofpital people, about five drachms of laudanum were taken by one of the patients. I did not fee him for feveral hours after he had taken it; he was then feemingly dying; pulfe low and weak, the heart labouring much, fkin cold, and covered with a clammy fweat. Though I had not the fmalleft hopes of fuccefs from any means that could be devifed for faving him, I diluted about two ounces of elixir of vitriol with a fufficient quantity of water, and, by means of a tea-pot, forced down his throat the greateft part of it.

What would almoft appear incredible, within half an hour he was able to fpeak, and called for victuals. I had once witneffed a fatal inftance of poifon by laudanum, the quantity taken not half an ounce; though medical aid was at hand, and the patient, a ftout woman, had lived upwards of fix hours after fhe had fwallowed it. The vitriolic acid was not tried in this cafe.

Dr. Trotter, a phyfician far above either my praife or cenfure, has propofed bleeding as a remedy in fome cafes of ague, which I am confident will will never be found neceffary. I once faw a foldier who was fubject to ague, taken ill with it the day after he was blooded. I can, from experience in upwards of four hundred cafes of ague, recommend the following mode of cure as certainly efficacious;

℞ Pulv. cinchonæ flavæ, drachm. x.

Tinct. opii. drachm. i.

Pulv. pimento, vel pip. nigr. drachm. i. fs. M.

Of this, either put into a bottle of water, or made into an electuary, the patient is directed to take a dofe as foon as the cold ftage comes on; and which he is to repeat in fuch quantities, and at fuch intervals, fo as to finifh the whole within twenty-four hours. After the medicine has been taken as here directed, I have very feldom met relapfes, or found a repetition of it neceffary. An emetic given, in a tertian, about an hour before the fit is expected, will in general remove it entirely. Thirty drops of laudanum given before the fit, will commonly produce the fame effect. But in the quotidian or quartan, both will be, for the moft part, found ineffectual. In the cure of agues the diet fhould be low, and every article containing alcohol carefully refrained from.

I am, Gentlemen,

ASHBURTON, Your humble Servant,

24th, *Auguft*, 1799. A. HUGGAN.

---

*To the Editors of the Medical and Phyfical Journal.*

